# Activation of somatodendritic 5-HT_1A_ autoreceptors reduces the acquisition and expression of cued fear in the rat fear-potentiated startle test

**DOI:** 10.1007/s00213-018-5124-0

**Published:** 2018-12-12

**Authors:** Yulong Zhao, Elisabeth Y. Bijlsma, Freija ter Heegde, Monika P. Verdouw, J. Garssen, Adrian Newman-Tancredi, Lucianne Groenink

**Affiliations:** 1 Division of Pharmacology, Utrecht Institute for Pharmaceutical Sciences (UIPS), Faculty of Science, Utrecht University, Utrecht, The Netherlands; 2 Neurolixis Inc., Dana Point, CA USA; 3 Brain Center Rudolf Magnus (BCRM), UMC Utrecht, Utrecht, The Netherlands

**Keywords:** Anxiety, Fear conditioning, Cued fear, Contextual anxiety, serotonin_1A_ receptor, Fear learning, Biased agonist, Fear-potentiated startle, Rats

## Abstract

**Rationale:**

Fear conditioning is an important factor in the etiology of anxiety disorders. Previous studies have demonstrated a role for serotonin (5-HT)_1A_ receptors in fear conditioning. However, the relative contribution of somatodendritic 5-HT_1A_ autoreceptors and post-synaptic 5-HT_1A_ heteroreceptors in fear conditioning is still unclear.

**Objective:**

To determine the role of pre- and post-synaptic 5-HT_1A_ receptors in the acquisition and expression of cued and contextual conditioned fear.

**Methods:**

We studied the acute effects of four 5-HT_1A_ receptor ligands in the fear-potentiated startle test. Male Wistar rats were injected with the 5-HT_1A_ receptors biased agonists F13714 (0–0.16 mg/kg, IP), which preferentially activates somatodendritic 5-HT_1A_ autoreceptors, or F15599 (0–0.16 mg/kg, IP), which preferentially activates cortical post-synaptic 5-HT_1A_ heteroreceptors, with the prototypical 5-HT_1A_ receptor agonist R(+)8-OH-DPAT (0–0.3 mg/kg, SC) or the 5-HT_1A_ receptor antagonist WAY100,635 (0–1.0 mg/kg, SC).

**Results:**

F13714 (0.16 mg/kg) and R(+)-8-OH-DPAT (0.03 mg/kg) injected before training reduced cued fear acquisition. Pre-treatment with F15599 or WAY100,635 had no effect on fear learning. In the fear-potentiated startle test, F13714 (0.04–0.16 mg/kg) and R(+)-8-OH-DPAT (0.1–0.3 mg/kg) reduced the expression of cued and contextual fear, whereas F15599 had no effect. WAY100,635 (0.03–1.0 mg/kg) reduced the overall startle response.

**Conclusions:**

The current findings indicate that activation of somatodendritic 5-HT_1A_ autoreceptors reduces cued fear learning, whereas 5-HT_1A_ receptors seem not involved in contextual fear learning. Moreover, activation of somatodendritic 5-HT_1A_ autoreceptors may reduce cued and contextual fear expression, whereas we found no evidence for the involvement of cortical 5-HT_1A_ heteroreceptors in the expression of conditioned fear.

## Introduction

Fear conditioning is considered an important factor in the etiology of anxiety disorders (Tinoco-Gonzalez et al. 2015). Several fear conditioning processes, including acquisition and expression, have been implicated in the pathogenesis of anxiety disorders (Lissek et al. 2008; Duits et al. 2015; Careaga et al. 2016; Jasnow et al. 2017) The fear-potentiated startle test, which is based on classical fear conditioning, has proven a valuable tool to study mechanisms involved in the acquisition and expression of conditioned fear in both rats and humans (Davis 1986; Grillon 2008). During a typical acquisition training, an organism learns to associate an aversive unconditioned stimulus, such as a shock, with a previously neutral conditioned stimulus, such as a cue light (Davis et al. 1993). Consequently, during testing, the response to startle-eliciting stimuli in the presence of the conditioned stimulus will be potentiated (Brown et al. 1951; Davis et al. 1979). Previous studies have shown that anxiolytic drugs can reduce the cued fear-potentiated startle response in rats (Davis et al. 1993; Nevins and Anthony 1994; Joordens et al. 1998) and human beings (Grillon et al. 2003; Hermans et al. 2006; Grillon 2008). Besides the discrete cue conditioning, the environment in which subjects are trained to associate cue with shock will come to elicit unpredictable threat (McNish et al. 1997). This conditioned contextual fear, also known as background anxiety, is associated with an increase in the non-cued startle response (Guscott et al. 2000; Groenink et al. 2008). The increase in the non-cued startle response in the presence of contextual cues has also been shown to be sensitive to anxiolytic drugs in rats (Santos et al. 2006; Almada et al. 2009; Ayers et al. 2011; Zhao et al. 2018b) and human subjects (Baas et al. 2002; Grillon et al. 2006).

Previous studies have demonstrated a critical role for the amygdala and hippocampus in fear-potentiated startle both during acquisition and expression (reviewed in Fendt et al. 1997; Lehmann et al. 2010; Wotjak and Pape 2013), whereas the pre-frontal cortex may modulate the expression of cued and contextual fear expression via projections to the amygdala (Wotjak and Pape 2013; Ferreira and Nobre 2014; Almada et al. 2015). In addition, a pathway from the central amygdala via the lateral periaqueductal gray to the caudal pontine reticular nucleus is involved in the expression of fear-potentiated startle (Fendt et al. 1997), whereas the bed nucleus of the stria terminalis (BNST) is strongly implicated in the regulation of contextual fear responses (Walker and Davis 1997).

Over the years, several studies have indicated that conditioned startle responses and conditioned freezing responses in rodents are modulated by different circuitries (McNish et al. 2000), including distinct involvement of the serotonergic system therein (Santos et al. 2006; Silva et al. 2002, 2004). Given that comparable fear-potentiated startle paradigms are being used in human and animal studies, we used the fear-potentiated startle paradigm to study the role of 5-HT_1A_ receptors in conditioned fear, since this may aid to the translational value of our studies.

Accumulating evidence suggests that serotonergic neurotransmission is altered in anxiety disorders. 5-HT_1A_ receptors may modulate anxiety in both its normal and pathological forms (Altieri et al. 2013; Popova and Naumenko 2013; Stiedl et al. 2015). A recent paper from Baas and Heitland (2015) suggests that a 5-HT_1A_ receptor polymorphism (rs6295) is associated with enhanced contextual anxiety, which further supports a role for human 5-HT_1A_ receptors in anxiety disorders and is in line with pre-clinical studies showing that 5-HT_1A_ receptor deletion enhanced the fear response to contextual cues in mice (Klemenhagen et al. 2006). However, the influence of 5-HT_1A_ receptors is complex, because they function both as somatodendritic autoreceptors and post-synaptic heteroreceptors. Pre-synaptic 5-HT_1A_ receptors are expressed on serotonergic cell bodies in the raphe nuclei and inhibit serotonin release by reducing the firing rate of serotonergic neurons. Post-synaptic 5-HT_1A_ receptors are located in multiple brain regions, including the cortex, amygdala, hippocampus, septum, and hypothalamus (Pazos and Palacios 1985; Chalmers and Watson 1991). These post-synaptic receptors are associated with control of emotions such as anxiety and fear (Kia et al. 1996; Meneses and Perez-Garcia 2007). Activation of 5-HT_1A_ receptors in these different brain regions exerts different effects on fear-potentiated startle. For example, pre-training injection of R(+)-8-OH-DPAT, a 5-HT_1A_ receptor agonist, into the median raphe nucleus reduced the acquisition of fear-potentiated startle, but did not influence the expression of fear-potentiated startle (Silva et al. 2002, 2004). Similarly, infusion of flesinoxan, a 5-HT_1A_ receptor agonist, into the median or dorsal raphe nucleus had no effect on the expression of fear-potentiated startle. Infusion of flesinoxan in the central amygdala, however, did reduce the expression of fear-potentiated startle (Groenink et al. 2000). Together, these brain region-specific studies suggest that pre-synaptic and post-synaptic 5-HT_1A_ receptors could differentially affect fear-potentiated startle and the acquisition and expression thereof.

In the present study, we applied the pharmacological approach of biased agonism to further study pre- versus 5-HT_1A_ post-synaptic receptor involvement in cued and contextual fear. Biased agonism denotes a phenomenon in which agonists may more efficaciously recruit one intracellular signaling pathway over another (Mannoury la Cour et al. 2006; Kenakin 2007; Millan et al. 2008). 5-HT_1A_ receptors are G protein-coupled receptors (GPCRs), which may activate different signaling pathways, including cyclic adenosine monophosphate (cAMP), inwardly rectifying K^+^ current (GIRK) and phosphorylation of extracellular signal-regulated kinase (ERK) via coupling to Gαi/Gαo proteins (Hamon et al. 1990; El Mestikawy et al. 1991). The interaction of 5-HT_1A_ receptors with the different GPCR pathways is to a certain extent brain region specific. In the raphe nuclei, for example, 5-HT_1A_ receptors have been shown to interact with Gαi3, whereas in the cerebral cortex, they may act via both Gαo and Gαi3 proteins (for review, see Altieri et al. 2013).

Several lines of evidence indicate that the biased agonists F15599 and its chemical congener F13714 target different 5-HT_1A_ receptor-mediated intracellular signaling pathways, thus allowing distinct pharmacological targeting of 5HT_1A_ receptor subpopulations (Newman-Tancredi 2011). For example, a c-Fos induction experiment showed that a low dose of F15599 (0.16 mg/kg IP) preferentially elicited c-Fos expression in the prefrontal cortex rather than in other brain regions (Newman-Tancredi et al. 2009). Similarly, a microdialysis study showed that doses up to 0.16 mg/kg IP of F15599 preferentially elicited dopamine release in frontal cortex, a post-synaptic 5-HT_1A_ receptor response, whereas higher doses reduced serotonin release, a 5-HT_1A_ autoreceptor-mediated response (Llad*ó*-Pelfort et al. 2010). Conversely, F13714 preferentially modulated ERK1/2 phosphorylation via 5-HT1A autoreceptor mechanisms and reduced 5-HT release at lower doses than those that increased cortical DA release (Buritova et al. 2009; Newman-Tancredi 2011). A pharmacoMRI brain imaging study showed that BOLD (Blood Oxygen Level Dependence) signal was specifically produced in frontal cortex by F15599 (0.16 mg/kg IP), whereas F13714 (0.04 mg/kg IP) and 8-OH-DPAT (0.32 mg/kg IP) elicited different patterns of brain activation including subcortical and other brain regions (Becker et al. 2016).

Behavioral studies showed anxiolytic actions for both F15599 and F13714 in a conditioned ultrasonic vocalization test and elevated plus maze, whereas both drugs were without effect in the Vogel lick test (Assié et al. 2010; Jastrzębska-Więsek et al. 2018). In cognitive tasks, these drugs had opposite actions, with F15599 facilitating learning and F13714 impairing reversal and spatial learning (Depoortère et al. 2010; van Goethem et al. 2015).

In the present study, the 5-HT_1A_ biased receptor agonists F13714 and F15599, the prototypical 5-HT_1A_ receptor agonist R(+)-8-OH-DPAT, and the 5-HT_1A_ receptor antagonist WAY100,635 were used to investigate the role of pre- and post-synaptic 5-HT_1A_ receptors in the acquisition and expression of cued and contextual fear in the rat fear-potentiated startle test. Based on the above, we hypothesized that fear acquisition would be reduced by somatodendritic 5-HT_1A_ receptor activation and facilitated by post-synaptic 5-HT_1A_ receptor activation. We further hypothesized that activation of post-synaptic 5-HT_1A_, but not pre-synaptic 5-HT_1A_ receptors, would reduce fear expression.

## Materials and methods

### Animals and housing

A total of 336 male rats (Wistar HsdCpd WU, Harlan Laboratories, Horst, Netherlands) were tested in the present study. For the acquisition study, 288 rats were used. Each drug was tested in 72 rats, divided over two cohorts of 36 rats. For the expression study, 48 rats were used. Each drug was tested in 12 rats using a balanced crossover within-subjects design (aka Latin square design). Sample size calculations for both experiments were based on previous studies in our laboratory (Bijlsma et al. 2015). A methodological study from our lab showed that male and female rats do not differ in their fear-potentiated startle response (Zhao et al. 2018a). The present study was therefore only performed in male rats to reduce the total number of animals needed.

Upon arrival in the laboratory, rats (6 weeks of age) were randomly allocated to the cages (four per cage), with controlled temperature (22 °C ± 2), humidity (55% ± 15), and light (lights on from 6 AM to 6 PM). Food and water were available ab libitum in the home cages. Rats were handled daily during the 1-week acclimation period. Experiments were carried out during the light phase of the day-night cycle between 8 AM and 5 PM. This allowed us to relate the effects of R(+)-8-OH-DPAT and WAY100,635 to previous work in our laboratory. Animal care and experimental procedures were conducted in compliance with the Dutch Experiments on Animals Act (EAA, amended 1996) and European regulations (guideline 86/609/EEC). All experiments were approved by the Committee on Care and Use of Laboratory Animals (DEC) of Utrecht University, The Netherlands (assigned protocol numbers 2012.I.12.124, 2014.I.06.041, and 2014.I.09.072).

### Apparatus

Eight startle devices were used simultaneously (SR-lab, San Diego Instruments, San Diego CA, USA). The startle devices consisted of a Plexiglas cylinder (9-cm diameter and 20-cm length) placed on a Plexiglas base. Each startle device was placed in a ventilated sound attenuated cubicle. Cage movements were measured with a piezoelectric film attached to the Plexiglas base of the startle device. A calibration system (San Diego Instruments) was used to ensure comparable startle magnitudes across the eight devices throughout the experiment. Startle stimuli (95 dB, 100 dB, and 110 dB), consisting of 50-ms white noise bursts were presented through a piezoelectric tweeter situated 15.2 cm from the top of the cylinder. Startle amplitudes were sampled each millisecond during a period of 65 ms beginning at the onset of the startle stimulus. Throughout the experiment, a background noise of 70 dB was presented to drown out noises originating from outside the individual cubicles. The light stimulus was delivered by light in the ceiling situated 15.2 cm from the top of the cylinder. There was no background illumination in any of the experiments. During the training phase, these devices were equipped with a stainless-steel grid floor, which delivered a mild foot shock (0.6 mA). During the test phase after the fear acquisition, two contexts were used. The “same” context (identical to that in the training phase) was equipped with a grid floor whilst the “alternate” context was equipped with a PVC board floor and stripe wall to alter the context. In the fear expression studies, training and testing were performed in the same context.

### Drugs

F13174 (3-chloro-4-fluorophenyl-(-4-fluoro-4-{[(5-methyl-6-methylaminopyridin-2-ylmethyl)-amino]-methyl}-piperidin-1-yl-methanone) and F15599 (3-chloro-4-fluorophenyl-94-fluoro-{[(5-methyl-pyrimidin-2-ylmethyl)-amino]-methyl} piperidin-1yl)-methanone) were obtained via a non-commercial transfer agreement with Newman-Tancredi. F13714 and F15599 were administered intraperitoneally (IP) 60 min prior to fear-potentiated startle training and test. R(+)-8-OH-DPAT (R(+)-8-hydroxy-2-(di-n-propylamino) tetralin) was purchased from Tocris Bioscience (Bristol, UK) and administered subcutaneously (SC) 10 min before fear-potentiated startle training and test. WAY100,635 (N-[2-[4-(2-methoxyphenyl)-1-piperazinyl] ethyl]-N-(2-pyridinyl) cyclohexanecarboxamide trihydrochloride) was purchased from Abcam Biochemicals (Cambridge, UK) and injected (SC) 30 min before fear-potentiated startle training and test. Routes of administration were chosen based on most commonly used routes. Drug solutions were freshly prepared every day. All drugs were dissolved in 0.9% saline (vehicle) and administered in a volume of 2 ml/kg. The animals were weighed 1 day before the drug administration.

## Experimental procedures

### Habituation and allocation to experimental conditions

The habituation session allowed rats to acclimatize to the experimental setup and to measure their baseline startle responses. The session consisted of 30 50-ms white noise bursts with three different intensities: 95, 100, and 110 dB, presented in pseudorandom order. For the acquisition study, startle responses assessed during this habituation session were used to divide rats into a low and high startle group. From each of these two startle groups, rats were randomly allocated to drug, dose, and context condition, ensuring comparable average startle responses among different experimental groups. For the expression study, rats were randomly allocated to treatment conditions upon arrival in the laboratory.

### Experiment 1—fear acquisition study

Effects of F13714 (vehicle, 0.04, 0.16 mg/kg, IP), F15599 (vehicle, 0.04, 0.16 mg/kg IP), R(+)-8-OH-DPAT (vehicle, 0.03, 0.1 mg/kg, SC), and WAY100,635 (vehicle, 0.3, 1.0 mg/kg, SC) on the acquisition of fear-potentiated startle were measured during training and in the test 24-h post-training.

#### Fear-potentiated startle training

**Fig. 1:**
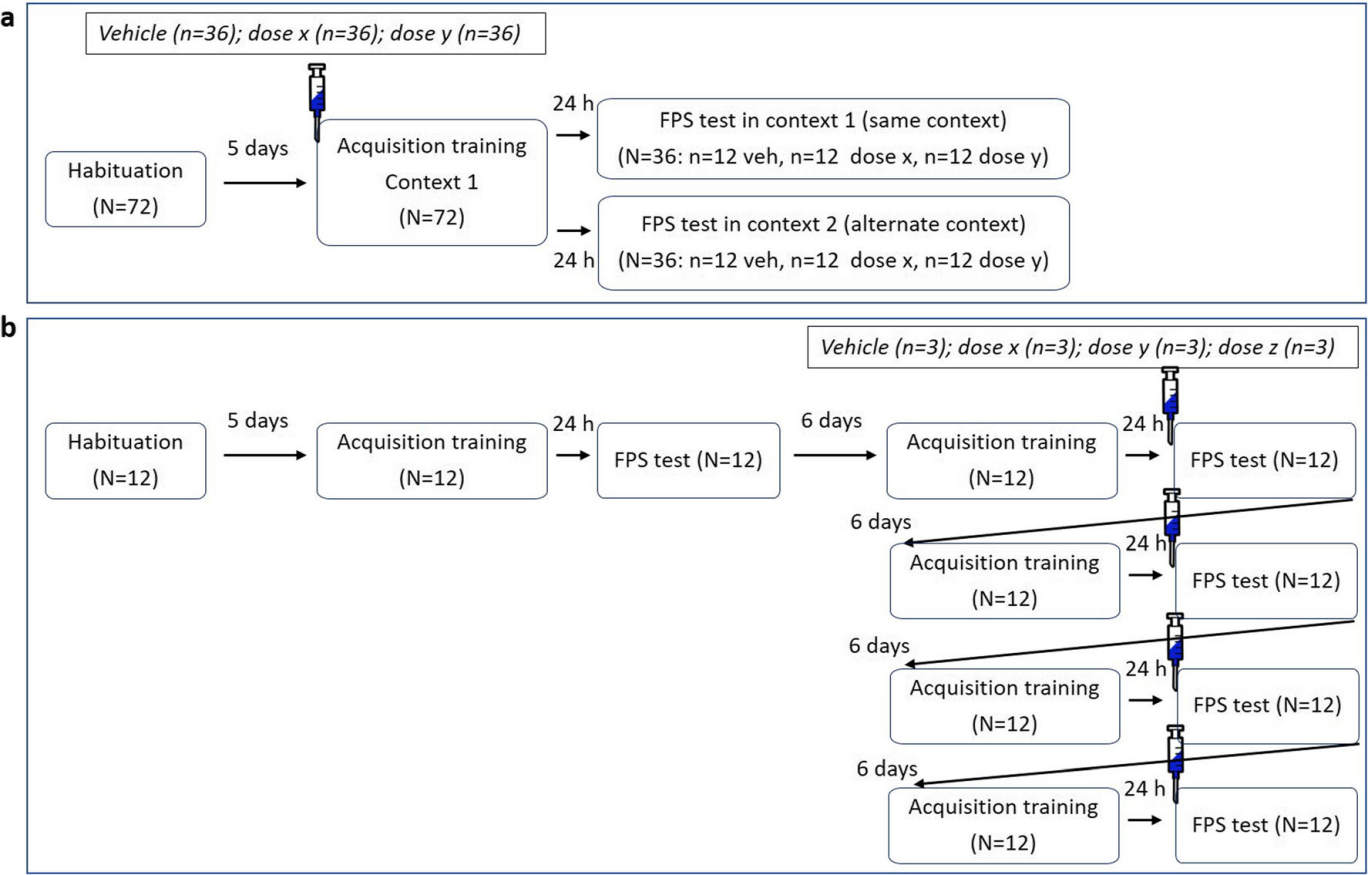
The experimental setup of experiment 1 (**a**) and experiment 2 (**b**) used to assess the effects of one compound. In experiment 2, each animal received each of four doses (including vehicle) of a compound once following a Latin square design, as indicated by the four syringes in figure **b**. FPS fear-potentiated startle

Prior to the training session, naïve rats were injected with the drug and dose they had been randomly allocated to (see Fig. 1). After the appropriate injection test interval, rats were placed in the startle device. Following an acclimatization period of 5 min, rats were presented with ten startle stimuli of 100 dB at a 30-s inter-stimulus interval (ISI) for rapid startle habituation. Next, rats were presented with ten light-shock pairings consisting of a 3700 ms light co-terminating with a 500 ms, 0.6 mA foot shock at an average interval of 4 min (range 3–5 min). The acquisition process was measured by presenting startle stimuli of 100 dB across the acquisition session in the absence and presence of the cue light. Before the first light-shock pairing rats were exposed to four startle stimuli (100 dB), half of them delivered in darkness (non-cued trials), the other half delivered during the last 50 ms of a 3250-ms light period (cued trials). Between every light-shock pairing rats were exposed to two startle stimuli, one non-cued trial and the other cued trial. All rats were trained in the same context.

#### Fear-potentiated startle test 24 h after training

The day after the training session, expression of fear-potentiated startle in rats was assessed. During this test session, no drugs were administered. Rats from each previous dose group were randomly allocated to one of two test contexts. One half of rats were tested in the same context as during training, the other half were tested in the alternate context. After an acclimation period of 5 min, ten startle stimuli of 110 dB were presented for habituation purposes (ISI 30 s), followed by 60 startle stimuli at an ISI of 30 s, 20 each at 95, 100, and 110 dB. Half of the 60 startle stimuli were presented during the last 50 ms of a 3250-ms light-on period (cued trials), the other half were delivered in darkness (non-cued trials). These six different trial types were presented in a balanced, irregular order during the test. Responses to the first ten habituation trials were not included in calculations of the mean startle response.

### Experiment 2—fear expression study

Effects of F13714 (vehicle, 0.01, 0.04, 0.16 mg/kg), F15599 (vehicle, 0.01, 0.04, 0.16 mg/kg), R(+)-8-OH-DPAT (vehicle, 0.03, 0.10, 0.30 mg/kg), and WAY100,635 (vehicle, 0.1, 0.3, 1.0 mg/kg) on the expression of fear-potentiated startle were measured. Each drug was tested in a separate group of animals, of which each rat received each dose once.

#### Fear-potentiated startle training and baseline test

Rats received training without injection of any drugs. During this session, rats were presented with ten light-shock parings with an average interval of 4 min (range, 3–5 min). A 0.6-mA shock was presented during the last 500 ms of the 3700-ms light period. The day after this training, a fear-potentiated startle test was performed to determine the baseline of fear-potentiated startle level (see Fig. 1). The test session characteristics were the same as in experiment 1.

#### Expression of fear-potentiated startle with drugs

One week after the baseline fear-potentiated startle test, rats received another training session, followed by a fear-potentiated startle test 24 h later. During the following 3 weeks, training and test procedures as described for experiment 2 were repeated once a week. Before each test, rats were injected with one of four doses of the drug (including vehicle) following a Latin square design, with all possible drug orders occurring only once, as shown in Fig. 1.

### Statistical analysis

Analyses were performed on absolute values of mean startle amplitudes. For each trial type, absolute peak values of mean startle amplitudes were averaged over the three noise intensities (95 dB, 100 dB, 110 dB).

In experiment 1, drug effects on fear-potentiated startle measured 24-h post-training were analyzed using a repeated measures analysis of variance (ANOVA) with cue (2 levels, non-cued and cued trials) as a within-subject factor and dose (3 levels, vehicle and two doses of each drug) and context (2 levels: same and alternate) as between-subjects factors. For this between-subjects design, ANOVA post hoc comparisons and drug effects on foot shock reactivity and percent fear potentiation were analyzed using one-way ANOVA followed by Dunnett’s two-tailed *t* test. Three rats were excluded from part of the analyses because of data sampling problems (*n* = 2 F15599 study, vehicle group, alternate context; *n* = 1 WAY100,635 study, vehicle group, same context). Eighteen rats in the WAY100635 group were accidentally tested in the same context, instead of in the alternate context. These rats were added to the “same context” group. Percent fear potentiation was calculated as follows: ([cued trials − non-cued trials]/non-cued trials) × 100%), and analyzed in case of a significant dose × cue interaction in the absence of main drug effects on cued and non-cued trials.

In experiment 2, drug effects on expression of fear-potentiated startle were analyzed using repeated measures ANOVA with cue (2 levels, non-cued and cued trials) and dose (4 levels, vehicle and three doses per drug) as within-subject factors. For this within-subject design, comparisons between drug doses and corresponding vehicle were made by simple contrasts.

A Greenhouse-Geisser correction was applied if sphericity assumptions were violated. The level of significance for all analyses was set at *p* < 0.05.

## Results

### Experiment 1. Fear acquisition

#### Effect of F13714 on fear acquisition

**Fig. 2:**
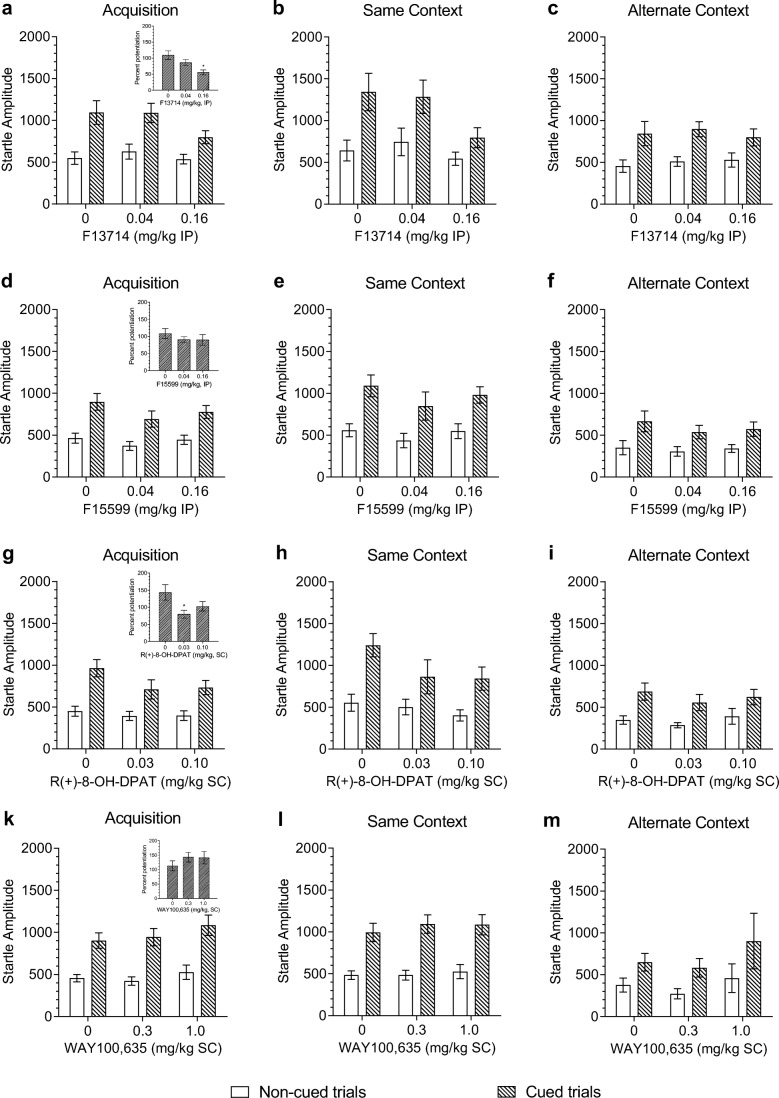
The effect of treatment with the 5-HT_1A_ receptor ligands F13714 (a, b, c), F15599 (d, e, f), R(+)-8-OH-DPAT (g, h, j), or WAY100,635 (k, l, m) on the acquisition of the fear-potentiated startle measured 24-h post-training. Left panels show the drug effects independent of context. a F13714 (*n* = 24 for each dose group), d F15599 (vehicle *n* = 23; 0.04 mg/kg F15599 *n* = 24; 0.16 mg/kg F15599 *n* = 24), g R(+)-8-OH-DPAT (*n* = 24 for each dose group), and j WAY100,635 (vehicle *n* = 23, *n* = 24 for the other dose groups)). Insets in a, d, g, and j show the effect of F13714, F15599, R(+)-8-OH-DPAT, and WAY100,635 on percent fear potentiation. Middle and right panels depict the drug effects obtained in the same (b) and alternate context (c) for F13714 (*n* = 12 for each dose group, in each context), F15599 (e same context, *n* = 12 for each dose group, f alternate context *n* = 10 for vehicle, *n* = 12 for 0.04 mg/kg, *n* = 12 for 0.16 mg/kg), R(+)-8-OH-DPAT (*n* = 12 for each dose group, in h same and i alternate context), and WAY100,635 (k same context, vehicle *n* = 17, other dose groups *n* = 18, and l alternate context (*n* = 6 for each dose group)). Data are shown as mean startle amplitudes (± SEM) to cued (hatched bars) and non-cued trials (open bars) or mean percent fear potentiation (± SEM, inset in a, d, g and k). *p < 0.05 compared to the vehicle condition. In all 4 experiments, a significant difference between cued and non-cued trials was induced. These significant effects are not depicted in the graphs

Fear acquisition training resulted in significant expression of fear potentiation 24 h later (cue effect *F*_1,66_ = 173, *p <* 0.001). The response to cued and non-cued trials in the test was dependent on prior treatment with F13714 (cue × dose *F*_2,66_ = 4.9, *p =* 0.01, see Fig. 2a), but independent of the context in which the rats were tested (cue × dose × context *F*_2,66_ = 1.3, *p =* 0.3; dose × context *F*_2,66_ < 1, see Fig. 2b, c). Further analyses showed that F13714 had no significant effect on the magnitude of the startle response to cued or non-cued trials per se (dose effect, cued trials *F*_2,69_ = 2.24, *p =* 0.11; non-cued trials *F*_2,69_ *<* 1), but percent fear potentiation was significantly lower in rats that had received treatment with 0.16 mg/kg F13714 before acquisition training (Dunnett’s two-tailed *t* test after significant dose effect *F*_2,69_ *=* 6.7, *p =* 0.002, see insert in Fig. 2a). F13714 did not alter the response to foot shock during training (Table 1; dose effect *F*_2,69_ *<* 1).

**Table 1:** Drug effects on foot shock reactivity during fear acquisition training

Drug	Dose (mg/kg)	Foot shock (startle amplitude)
F13714	Vehicle	241 ± 31.8
	0.04	228 ± 22.4
	0.16	208 ± 19.6
F15599	Vehicle	289 ± 30.7
	0.04	200 ± 16.3*
	0.16	242 ± 25.8
R(+)-8-OH-DPAT	Vehicle	279 ± 21.4
	0.03	229 ± 31.3
	0.1	212 ± 22.9
WAY100,635	Vehicle	316 ± 30.7
	0.3	257 ± 21.8
	1.0	229 ± 18.6*

Data are presented as mean startle amplitude ± SEM. *p < 0.05 relative to vehicle control

**Fig. 3:**
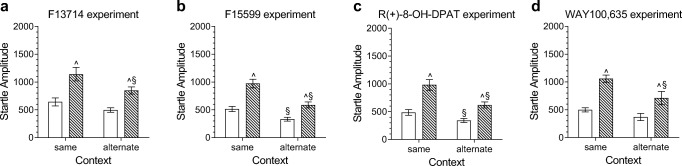
The effect of acquisition training on the startle response to cued (hatched bars) and non-cued (open bars) trials as measured in the same and alternate context 24-h post-training. **a** F13714 experiment (*n* = 36 for each context); **b** F15599 experiment (same context *n* = 36, alternate context *n* = 34); **c** R(+)-8-OH-DPAT (*n* = 36 for each context); and **d** WAY100,635 (same context *n* = 54, alternate context *n* = 18). Data are shown as mean startle amplitudes (± SEM). For each experiment, data are collapsed over treatment condition. § *p* < 0.05 compared to the corresponding condition in the same context. ^ *p* < 0.05 compared to response to non-cued trials

The startle response to cued and non-cued trials was dependent on the context in which the rats were tested (cue × context *F*_1,66_ = 5.3, *p* = 0.025). As shown in Fig. 3a, fear potentiation was significantly smaller in the alternate than in the same context, whereas the response to non-cued trials did not differ significantly between contexts (two-tailed Student’s *t* test *p* = 0.03, and *p* = 0.1 respectively). The overall startle response was significantly higher in the same than in the alternate context (*F*_2,66_ = 4.4, *p* = 0.04).

#### Effect of F15599 on fear acquisition

Fear acquisition training was successfully induced as indicated by a significantly higher startle response to cued than non-cued trials 24 h post-training (cue effect *F*_1,64_ = 155, *p* < 0.001). Treatment with F15599 before the acquisition training did not alter the level of fear potentiation (cue × dose *F*_2,64_ = 1.2, *p* = 0.3, see Fig. 2d), percent fear potentiation (*F*_2,67_ < 1) or the overall startle response on the test day (dose effect *F*_2,64_ = 1.0, *p* = 0.4). Furthermore, prior treatment with F15599 did not alter the effect of context on the fear-potentiated startle response (cue × dose × context *F*_2,64_ < 1; dose × context *F*_2,64_ < 1, see Fig. 2e, f). Treatment with 0.04 mg/kg F15599, but not 0.16 mg/kg, significantly reduced the response to foot shock (see Table 1; dose effect *F*_2,68_ = 3.2, *p* = 0.045).

The overall startle response was significantly higher in the same than in the alternate context (context *F*_1,64_ = 13.8, *p* < 0.001), as was the level of fear potentiation (cue × context *F*_1,64_ = 11.9, *p* < 0.001). Post hoc two-tailed Student’s *t* tests further showed that both the response to cued and non-cued trials were significantly smaller in the alternate context (cued trials *p* < 0.001; non-cued trials *p* = 0.004, see Fig. 3b).

#### Effect of R(+)-8-OH-DPAT on fear acquisition

Acquisition training resulted in significant fear-potentiated startle 24 h later (cue effect *F*_1,66_ = 131.1, *p* < 0.001). Treatment with R(+)-8-OH-DPAT during fear acquisition training differentially affected the response to cued and non-cued trials (cue × dose *F*_2,66_ = 3.4, *p* = 0.04, see Fig. 2g), but this drug effect was independent of the context in which animals were tested (cue × dose × context *F*_2,66_ = 1.2, *p* = 0.3; dose × context *F*_2,66_ < 1, see Fig. 2h, i). Further analyses showed that pre-treatment with R(+)-8-OH-DPAT had no effect on the response to cued or non-cued trials per se (dose effect cued trials *F*_2,69_ = 1.9, *p* = 0.15; non-cued trials *F*_2,69_ < 1). Post hoc analysis however showed that R(+)-8-OH-DPAT pre-treatment reduced the percent fear potentiation at the lowest dose relative to vehicle treatment (dose effect *F*_2,69_ = 3.7, *p* = 0.03, see insert in Fig. 2g). R(+)-8-OH-DPAT did not alter the response to foot shock (see Table 1; *F*_2,69_ = 1.9, *p* = 0.16).

Both the overall startle response and the level of fear potentiation were significantly stronger in the same context than in the alternate context (context effect *F*_1,66_ = 9.3, *p* = 0.003, cue × context *F*_1,66_ = 10.1, *p* = 0.002). Post hoc independent two-tailed Student’s *t* tests further showed that both the responses to cued and non-cued trials were significantly lower in the alternate context (cued trials *p* = 0.002; non-cued trials *p* = 0.025, see Fig. 3c).

#### Effect of WAY100,635 on fear acquisition

Acquisition training resulted in significant expression of fear-potentiated startle in the test 24 h later (cue effect *F*_1,65_ = 112, *p* < 0.001). The level of fear potentiation was independent of prior treatment with WAY100,635 (cue × dose *F*_2,65_ < 1, see Fig. 2k), as was the percent of fear potentiation (dose effect *F*_2,68_ < 1). Pre-treatment with WAY100,635 also had no effect on the mean startle response (dose effect *F*_2,65_ < 1). Effects were independent of the context in which expression of fear-potentiated startle was measured (cue × dose × context *F*_2,65_ < 1; dose × context *F*_2,65_ < 1, see Fig. 2l, m). Rats treated with the highest dose of WAY100,635 showed a significantly lower response to foot shock than vehicle-treated rats during training (see Table 1; *F*_2,69_ = 3.3, *p* = 0.04).

The overall startle response and the level of fear potentiation were significantly higher in the same context than in the alternate context (context effect *F*_1,65_ = 5.6, *p* = 0.02; cue × context *F*_1,65_ = 6.6, *p* = 0.01, see Fig. 3d). Further analysis showed that the response to cued trials was significantly smaller in the alternate context, whereas responses to non-cued trials did not differ significantly between contexts (cued trials *p* = 0.01; non-cued trials *p* = 0.09, see Fig. 3d).

### Experiment 2: expression of fear-potentiated startle

In all experiments, fear-potentiated startle was successfully induced (cue effect, F13714 test *F*_1,11_ = 73.2, *p* < 0.001; F15599 test *F*_1,11_ = 88.3, *p* < 0.001; R(+)-8-OH-DPAT test *F*_1,11_ = 24.2, *p* < 0.001; WAY100,635 test *F*_1,11_ = 69.1, *p* < 0.001).

#### Effect of F13714 on expression of fear-potentiated startle

**Fig. 4:**
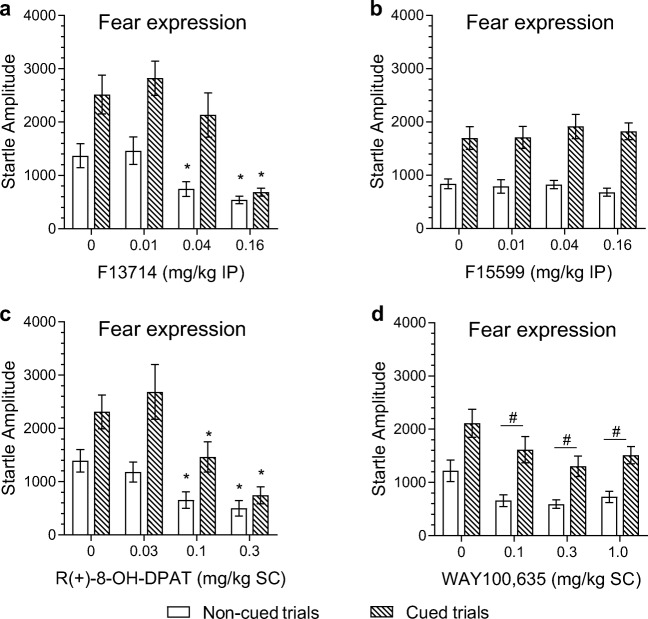
The effects of F13714 (a), F15599 (b), R(+)-8-OH-DPAT (c), and WAY100,635 (d) on fear expression in the fear-potentiated startle test. Data are shown as mean startle amplitudes (± SEM) in response to the non-cued (filled circles) and cued trials (open circles) (*n* = 12 for each dose condition). * *p* < 0.05 compared to vehicle-treated rats. # *p* < 0.05 represents a significant difference in overall startle response compared to the vehicle condition. In all 4 experiments, a significant difference between cued and non-cued trials was induced. These significant effects are not depicted in the graphs

As shown in Fig. 4a, F13714 significantly reduced the startle response in the fear-potentiated startle test (dose effect, *F*_3,33_ = 22.5, *p* < 0.001). This drug effect was dependent on trial type (cue × dose, *F*_3,33_ = 9.7, *p* = 0.001, *ε* = 0.65). F13714 had a stronger effect on the startle response to cued than to non-cued trials. The reduction in response to cued trials was significant at 0.16 mg/kg (dose effect, *F*_3,33_ = 20.2, *p* < 0.001; simple contrasts see Table 2), whereas treatment with 0.04 or 0.16 mg/kg F13714 significantly reduced the non-cued startle response relative to vehicle-treated rats (dose effect, *F*_3,33_ = 14.1, *p* = 0.001, *ε* = 0.43; simple contrasts see Table 2).

**Table 2:** Summary of simple contrasts results for drug effects on fear expression

	Non-cued trials		Cued trials	
F13714	*F*_1,11_	*p*	*F*_1,11_	*p*
0.01 mg/kg vs vehicle	1.5	0.3	1.2	0.3
0.04 mg/kg vs vehicle	21.0	0.001^*^	2.4	0.2
0.16 mg/kg vs vehicle	14.5	0.003^*^	32.4	< 0.001^*^
F15599^a^	Not applicable		Not applicable	
R(+)-8-OH-DPAT	*F*_1,11_	*p*	*F*_1,11_	*p*
0.03 mg/kg vs vehicle	2.1	0.2	0.6	0.4
0.10 mg/kg vs vehicle	8.0	0.02^*^	6.3	0.03^*^
0.30 mg/kg vs vehicle	12.4	0.005^*^	16.3	0.002^*^
WAY100,635^b^		*F*_1,11_	*p*	
0.10 mg/kg vs vehicle		8.0	0.02^*^	
0.30 mg/kg vs vehicle		8.2	0.02^*^	
1.00 mg/kg vs vehicle		8.9	0.01^*^	

^a^For F15599, no simple contrasts could be performed in the absence of a main dose or a dose × cue interaction effect

^b^For WAY100,635 simple contrasts were performed for overall mean startle response in the absence of a dose × cue interaction effect

*p < 0.05 compared to the vehicle condition

#### Effect of F15599 on expression of fear-potentiated startle

F15599 had no significant effect on the startle response in the fear-potentiated startle test (main effect dose *F*_3,33_ < 1; cue × dose interaction *F*_3,33_ = 1.2, *p* = 0.3, see Fig. 4b).

#### Effect of R(+)-8-OH-DPAT on expression of fear-potentiated startle

R(+)-8-OH-DPAT significantly reduced the startle response in the fear-potentiated startle test (dose effect, *F*_3,33_ = 9.4, *p* < 0.001). This effect was dependent on trial type (cue × dose, *F*_3,33_ = 5.2, *p* = 0.005). As shown in Fig. 4c, R(+)-8-OH-DPAT had a stronger effect on cued trials than on non-cued trials. Further analysis showed that R(+)-8-OH-DPAT significantly reduced the response to both cued (dose effect, *F*_3,33_ = 8.5, *p* < 0.001) and non-cued trials (dose effect, *F*_3,33_ = 8.4, *p* = 0.006, *ε* = 0.49) at 0.1 and 0.3 mg/kg (see Fig. 4c and Table 2).

#### Effect of WAY100,635 on expression of fear-potentiated startle

As shown in Fig. 4d, WAY100, 635 reduced the overall startle response in the fear-potentiated startle test (*F*_3,33_ = 6.57, *p* = 0.009, *ε* = 0.57). This effect was independent of trial type (cue × dose, *F*_3,33_ < 1). Simple contrasts showed that all doses of WAY100,635 (0.1, 0.3 and 1 mg/kg) significantly reduced the overall mean startle response relative to vehicle control (see Fig. 4d and Table 2).

## Discussion

In the present study, we investigated the role of 5-HT_1A_ receptors in fear conditioning by testing ligands with varying efficacy at different 5-HT_1A_ receptors in the rat fear-potentiated startle. We found that F13714, which pre-dominantly activates somatodendritic 5-HT_1A_ autoreceptors, impaired cued fear acquisition and reduced cued fear expression. Likewise, a low dose of R(+)-8-OH-DPAT reduced cued fear acquisition, whereas higher doses of R(+)-8-OH-DPAT were required to reduce cued fear expression. In contrast, selective activation of cortical post-synaptic 5-HT_1A_ receptors using F15599 did not alter the acquisition or expression of conditioned fear. Blockade of 5-HT_1A_ receptors with the 5-HT_1A_ receptor antagonist WAY100635, on the other hand, had no effect on fear acquisition but reduced the overall startle response in the fear expression test. Finally, none of the drugs appeared to alter contextual fear acquisition, since pre-treatment had no effect on non-cued trials. Also, all drug effects were independent of the context in which rats were tested 24 h later, indicating that the valence of the cue was not affected by drug-induced alterations in contextual fear acquisition.

### Acquisition of conditioned fear

#### Involvement of 5-HT_1A_ receptors in the acquisition of cued fear

We found that F13714 and R(+)-8-OH-DPAT reduced the acquisition of cued fear, whereas prior injection with F15599 or WAY100,635 had no effect on the cued fear response measured 24 h later.

F13714 is a highly selective 5-HT_1A_ receptor agonist that preferentially activates pre-synaptic 5-HT_1A_ receptors (Koek et al. 2001; Buritova et al. 2009; Newman-Tancredi et al. 2009). Also, effects of F13714 in the dose range tested can be blocked by the 5-HT_1A_ antagonist WAY100,635 (Buritova et al. 2009; van Goethem et al. 2015; De Boer and Newman-Tancredi 2016). Therefore, the finding that treatment with F13714 during acquisition training lowers the level of fear potentiation suggests that activation of somatodendritic 5-HT_1A_ autoreceptors inhibits cued fear learning. The effects obtained with R(+)8-OH-DPAT further confirm this finding. Indeed, the reduction in fear potentiation after prior treatment with R(+)-8-OH-DPAT followed a U-shaped curve. Electrophysiological, behavioral, and microdialysis studies in rats indicate that systemic administration of 8-OH-DPAT at low doses, up to approximately 0.3 mg/kg, pre-dominantly exerts effects through somatodendritic 5-HT_1A_ autoreceptors, whereas at higher doses, activation of post-synaptic 5-HT_1A_ receptors becomes more marked (Meller et al. 1990; Larsson et al. 1990; Hadrava et al. 1995; Schechter et al. 2005; Assié et al. 2005). Hence, our results suggest the involvement of somatodendritic 5-HT_1A_ autoreceptor activation in the reduced cued fear learning following treatment with R(+)-8-OH-DPAT. The U-shaped dose response curve suggests the presence of a counteraction effect via other receptors at the higher dose. Besides post-synaptic 5-HT_1A_ receptors, 5-HT_7_ receptors may be potential candidates for this, since 8-OH-DPAT exerts agonist activity at these receptors and it has been suggested that 5-HT_7_ receptors facilitate emotional learning (Bickmeyer et al. 2002; Stiedl et al. 2015). Together, the studies with F13714 and R(+)-8-OH-DPAT indicate that reduced serotonergic transmission through somatodendritic 5-HT_1A_ receptor activation attenuates cued fear learning.

In contrast, we found no evidence for involvement of post-synaptic 5-HT_1A_ receptors in cued fear learning. The absence of effect of F15599 on cued fear learning indicates that activation of cortical post-synaptic 5-HT_1A_ receptors is not sufficient to alter cued fear learning. The fact that the 5-HT_1A_ receptor antagonist, WAY100635, also had no effect on cued fear acquisition further suggests that activation of post-synaptic 5-HT_1A_ receptors is not required for cued fear learning. The absence of effect of WAY100635 on cued fear acquisition also indicates that enhanced synaptic availability of serotonin, through blockade of somatodendritic 5-HT_1A_ autoreceptors, does not alter cued fear acquisition. This is in line with a previous study in which we showed that enhanced serotonergic transmission through acute or chronic treatment with the SSRI paroxetine did not alter the acquisition—or expression—of fear-potentiated startle (Bijlsma et al. 2015).

Although 5-HT_1A_ receptors have been implicated in fear conditioning, their role in acquisition of cued or contextual conditioned fear in the fear-potentiated startle test has hardly been studied. The results obtained for cued fear acquisition using our pharmacological biased agonist approach are in line with those reported in the one previous paper that studied the role of 5-HT_1A_ receptors in the acquisition of cued fear-potentiated startle. Both infusion of 8-OH-DPAT in the median raphe nucleus as well as lesions of this nucleus reduced acquisition of cued fear in the rat fear-potentiated startle test (Silva et al. 2002).

Regarding the role of post-synaptic 5-HT_1A_ receptor activation in the acquisition of cued fear-potentiated startle, we are not aware of any previous study addressing this. Here, F15599 did not enhance cued fear learning as measured 24-h post-training. The absence of effect may seem to contrast the previously reported procognitive effects of F15599 (Depoortère et al. 2010; Horiguchi and Meltzer 2012; van Goethem et al. 2015), but emotional learning relies on different neuronal circuitries than reversal and spatial learning. Hence, different 5-HT_1A_ receptor subpopulations may be involved in the previously reported cognition tests compared with the present model.

The absence of effect of F15599 is not likely explained by the dose range in which the compound was tested. Apart from the effect on foot shock reactivity, the doses used in the present study have been shown to be active in a variety of behavioral studies. Both F15599 and F13714 suppressed immobility in the rat forced swim test and inhibited stress-induced ultrasonic vocalizations at doses of 0.04–0.16 mg/kg i.p. (Assié et al. 2010). In cognition tests, F15599 rescued working memory deficits at a dose of 0.16 mg/kg i.p., whereas F13714 (0.04 mg/kg i.p.) impaired performance (Depoortère et al. 2010). In hemiparkinsonian (i.e., unilaterally 6-OH-DA-lesioned) rats, F15599 and F13714 reversed L-DOPA-induced dyskinesias over a dose range of 0.02 to 0.16 mg/kg i.p. (Iderberg et al. 2015). Finally, in a rat model of aggressive behavior, F15599 reduced aggression from a dose of 0.125 mg/kg i.p. and F13714 was very potent, reducing aggression from a dose of 0.012 mg/kg i.p. (De Boer and Newman-Tancredi 2016). At doses above 0.16 mg/kg, F15599 has been shown to activate 5-HT_1A_ autoreceptors, in addition to cortical 5-HT_1A_ receptors, causing inhibition of 5-HT release (Llad*ó*-Pelfort et al. 2010).

#### Involvement of 5-HT_1A_ receptors in the acquisition of contextual fear

In all four experiments, the overall startle response in the alternate context was lower than that measured in the same context. This indicates that the procedure used successfully elicited conditioned contextual fear. We also consistently found that the magnitude of the response to cued trials was dependent on the context in which the cue was presented. In all experiments, the response to cued trials was significantly lower in the alternate context than in the same context. This finding supports the idea that the response to cued trials is not a simple addition of contextual anxiety and cued fear, but rather reflects an augmentation of cued fear in the presence of contextual cues (van Ast et al. 2012). The results further indicate, however, that none of the 5-HT_1A_ receptor ligands altered the acquired level of contextual conditioned fear. We found no drug effects on the non-cued startle response relative to the vehicle control conditions. Furthermore, the observed drug effects of F13714 and R(+)-8-OH-DPAT on fear potentiation were independent of the context in which the rats were tested. We are aware of only one paper studying acquisition of contextual fear in a startle paradigm. Borelli and co-workers (Borelli et al. 2005) showed that infusion of 8-OH-DPAT into the median raphe nucleus as well as lesions of this nucleus prior to training reduced the level of contextual conditioned fear in a startle paradigm. Since their design focused on contextual fear conditioning, no cues were presented during training. In a standard fear-potentiated startle training in which rats learn to associate cue with an aversive stimulus, the absence of the cue comes to serve as safety signal and may suppress fear levels, which could interfere with the acquisition of contextual fear (Ng et al. 2018). This difference in experimental setup may have contributed to the different outcome regarding the role of somatodendritic 5-HT_1A_ autoreceptor activation in contextual fear acquisition.

Finally, since for all four 5-HT_1A_ receptor ligands tested, anxiolytic and/or anxiogenic effects have been reported in one test or another (Groenink et al. 1996; Assié et al. 2010; Cryan et al. 2011; Jastrzębska-Więsek et al. 2018), it cannot be excluded that drugs may have altered the perceived aversiveness of the training conditions, and thereby the achieved level of fear potentiation. However, the response to foot shock during training does not support this notion. Both F15599 and WAY100,635 reduced the response to foot shock, but did not affect fear acquisition. Vice versa, F13714 and R(+)-8-OH-DPAT had no effect on foot shock reactivity but did impair cued fear learning.

Based on the above, we conclude that somatodendritic 5-HT_1A_ autoreceptors and 5-HT_1A_ heteroreceptors do not play a major role in the acquisition of conditioned contextual anxiety.

### Expression of conditioned fear

#### Involvement of 5-HT_1A_ receptors in the expression of cued fear

R(+)-8-OH-DPAT decreased the fear-potentiated startle response, which is in line with previous reports and indicative of an anxiolytic profile (Mansbach and Geyer 1988; Joordens et al. 1998; but see Davis et al. 1988). The linear dose-dependent effect of R(+)-8-OH-DPAT on cued fear differed from its U-shaped curve effect on fear acquisition and was obtained at higher doses (0.1, 0.3 mg/kg sc) than the reduction in cued fear acquisition (0.03 mg/kg sc). Yet, as outlined above, within the dose range tested, R(+)-8-OH-DPAT is thought to pre-dominantly exert effects through somatodendritic 5-HT_1A_ autoreceptors. Accordingly, like R(+)-8-OH-DPAT, F13714 also reduced the startle response to cued trials. Together these findings suggest that activation of somatodendritic 5-HT_1A_ autoreceptors may reduce the expression of cued fear. Our findings are not in line with fear-potentiated startle studies in which local infusion of 5-HT_1A_ receptor agonists into raphe nuclei or lesions of the median raphe nucleus proved without effect on cued fear (Groenink et al. 2000; Silva et al. 2004). It may be that selective targeting of 5-HT_1A_ receptor subpopulations through biased agonism yields a different net result on serotonergic transmission than local brain infusion, which may explain the diverging results. Systemic administration of F13714 or R(+)-8-OH-PDAT reduced vocalizations in the conditioned ultrasonic vocalization test (Remy et al. 1996; Assié et al. 2010), a response which is mediated through activation of somatodendritic 5-HT_1A_ autoreceptors, at least in the case of R(+)-8-OH-DPAT (Remy et al. 1996), suggesting that systemic administration of these ligands may indeed reduce anxiety through activation of pre-synaptic 5-HT_1A_ receptors.

In fact, it is generally assumed that anxiety is associated with enhanced serotonergic transmission (for review see Albert et al. 2014), which fits the idea that 5-HT_1A_ receptor agonists may exert their effect via somatodendritic 5-HT_1A_ autoreceptors. Whether this is also the case for the cued fear reduction in the fear-potentiated startle test requires further investigation. Despite their properties and the dose range in which F13714 and R(+)-8-OH-PDAT were tested, it may be that these drugs also exerted actions through post-synaptic 5-HT_1A_ heteroreceptors. This may initially seem counterintuitive, since somatodendritic 5-HT_1A_ receptor activation reduces serotonin availability in the synaptic cleft. But both activation of somatodendritic 5-HT_1A_ autoreceptors and activation of post-synaptic 5-HT_1A_ heteroreceptors could result in anxiolytic-like effects if activation of other, non-5-HT_1A_, post-synaptic 5-HT receptor subtypes induces anxiogenic-like effects. Interestingly, the 5-HT_2C_ receptor agonist mCPP does indeed enhance the fear-potentiated startle response (Bijlsma et al. 2010).

Thus, the finding that pre-synaptic 5-HT_1A_ receptor activation may reduce cued fear does not necessarily mean that post-synaptic 5-HT_1A_ receptors are not involved in the modulation of cued fear. Several studies have demonstrated that infusion of 5-HT_1A_ receptor agonists into the amygdala reduces the fear-potentiated startle response (Groenink et al. 2000; Ferreira and Nobre 2014). A more recent paper further suggested the involvement of post-synaptic 5-HT_1A_ receptors in the medial pre-frontal cortex in this effect (Ferreira and Nobre 2014). Infusion of 8-OH-DPAT—or serotonin—in the pre-limbic cortex reduced the fear-potentiated startle response in highly anxious rats. The authors proposed that the inhibitory action of 5-HT_1A_ heteroreceptor activation on glutamatergic pyramidal neurons in the pre-limbic cortex could decrease the excitation of basolateral amygdala neurons, eventually resulting in a reduced conditioned fear response (Ferreira and Nobre 2014). Using systemic administration of the biased 5-HT_1A_ receptor agonist, F15599 we could not confirm these findings. This might be explained by the fact that following systemic drug administration also 5-HT_1A_ receptors in other cortical sub regions are activated, which may have resulted in a different net outcome. The infralimbic cortex for instance may be involved in inhibition of fear, and 5-HT_1A_ receptor activation in this area may thus enhance fear expression (Sotres-Bayon and Quirk 2010).

The 5-HT_1A_ receptor antagonist WAY100,635 did not specifically alter the expression of cued fear, but rather reduced the overall startle response at all doses tested. Results obtained with WAY100,635 are very similar to previous findings from our laboratory (Joordens et al. 1997), in which WAY100,635 also reduced the overall startle response following a U-shaped dose response curve. At higher doses, WAY100,635 may also exert adrenergic α1 receptor antagonist actions at serotonergic neurons in the raphe. This could explain the U-shaped dose response curve, as outlined by Joordens and co-workers (Joordens et al. 1997). Alternatively, such an effect could be set about via indirect effects on the cholinergic system (Madjid et al. 2006).

#### Involvement of 5-HT_1A_ receptors in the expression of contextual fear

F13714 and R(+)-8-OH-DPAT both dose-dependently reduced the startle response to non-cued trials. This suggests the involvement of somatodendritic 5-HT_1A_ autoreceptors in the reduction of contextual fear expression. In an elegant series of experiments, Almada and co-workers showed that post-synaptic 5-HT_1A_ receptor activation following local infusion with 8-OH-DPAT in the rat dorsal hippocampus and pre-limbic cortex reduced conditioned contextual fear, as measured with the acoustic startle response (Almada et al. 2009, 2015). Infusion of 8-OH-DPAT in the median raphe nucleus on the other hand was without effect (Almada et al. 2009). Together those studies would imply the involvement of post-synaptic 5-HT_1A_ receptors in the reduction of conditioned contextual fear. However, in a standard fear-potentiated startle test, involving conditioning to both cue and context, infusion of 8-OH-DPAT in the pre-limbic cortex had no effect on contextual anxiety as reflected in the non-cued startle response, whereas the startle response to cued trials was reduced following infusion with 8-OH-DPAT (Ferreira and Nobre 2014). The latter finding is in line with the absence of effect of F15599 in the present study in which also the standard fear-potentiated startle test was used. As outlined above, this difference in fear training may have contributed to the different findings. Accordingly, further studies adding random shock control conditions to the design may help to disentangle the mutual interaction between discrete and contextual cues in fear conditioning and the involvement of 5-HT_1A_ receptors therein (Bijlsma et al. 2015).

A potentially other factor that could explain observed differences between our studies and those discussed above, is that those studies may have used S(−)-8-OH-DPAT or racemic 8-OH-DPAT rather than R(+)-8-OH-DPAT. Lejeune and co-workers showed that the effects of R(+)-8-OH-DPAT may differ from those of R(+)-8-OH-DPAT (Lejeune et al. 1997). This property of the compound was not specified in the papers (Borelli et al. 2005; Almada et al. 2009, 2015; Ferreira and Nobre 2014).

#### Concluding remarks

The use of biased agonists may be a valuable addition to the tools used to study the relative contribution of somatodendritic and post-synaptic 5-HT_1A_ receptors in anxiety, since drugs like R(+)-8-OH-DPAT lack specificity for 5-HT_1A_ receptor subpopulations and may diffuse to the raphe nuclei after infusion into forebrain regions hampering interpretation of results (Jolas et al. 1995). On the other hand, the dose range in which biased agonists exert their effect at particular receptor subpopulations and signaling pathways in vivo is not exactly known, which could be considered a potential limitation of the current study (although previous studies suggest a tenfold dose selectivity of F15599 for cortical 5-HT_1A_ receptors (Llad*ó*-Pelfort et al. 2010).

The current study indicates that 5-HT_1A_ receptors do not play a major role in the acquisition of conditioned contextual fear. Cued fear learning, on the other hand, is reduced by activation of somatodendritic 5-HT_1A_ autoreceptors. This study further indicates that activation of somatodendritic 5-HT_1A_ autoreceptors may reduce the expression of cued and contextual fear in the rat fear-potentiated startle test. Finally, we found no evidence for involvement of *cortical* post-synaptic 5-HT_1A_ heteroreceptors in the acquisition or expression of conditioned fear.
